# The Potential of Endophytic Fungi for Enhancing the Growth and Accumulation of Phenolic Compounds and Anthocyanin in Maled Phai Rice (*Oryza sativa* L.)

**DOI:** 10.3390/jof9090937

**Published:** 2023-09-16

**Authors:** Thanawan Gateta, Sabaiporn Nacoon, Wasan Seemakram, Jindarat Ekprasert, Piyada Theerakulpisut, Jirawat Sanitchon, Nakarin Suwannarach, Sophon Boonlue

**Affiliations:** 1 Department of Microbiology, Faculty of Science, Khon Kaen University, Khon Kaen 40002, Thailand; thanawan.gateta@kkumail.com (T.G.); n_sabaiporn@kkumail.com (S.N.); seemakram.w@gmail.com (W.S.); jindaek@kku.ac.th (J.E.); 2 Department of Biology, Faculty of Science, Khon Kaen University, Khon Kaen 40002, Thailand; piythe@kku.ac.th; 3 Salt-Tolerant Rice Research Group, Khon Kaen University, Khon Kaen 40002, Thailand; 4 Department of Agronomy, Faculty of Agriculture, Khon Kaen University, Khon Kaen 40002, Thailand; jirawat@kku.ac.th; 5 Center of Excellence in Microbial Diversity and Sustainable Utilization, Chiang Mai University, Chiang Mai 50200, Thailand; suwan.462@gmail.com

**Keywords:** *Trichoderma*, plant growth promoter, phytohormones, functional substances, black rice, antioxidant

## Abstract

Rice (*Oryza sativa* L.) is one of the most popular cereal crops, being consumed by almost half of the world’s population. Among several cultivars widely distributed in Thailand, Maled Phai is a Thai pigmented-upland rice with exceptionally high nutritional value and high demand in the local Thai market. This study aimed to investigate the feasibility of producing plant growth-promoting properties (PGP) and enhancing the accumulation of phytochemicals in Maled Phai rice seeds of endophytic fungi isolated from upland black rice. Among a total of 56 isolates, the 4 most effective PGP isolates were identified as *Trichoderma zelobreve* PBMP16, *Talaromyces pinophilus* PBMP28, *Aspergillus flavus* KKMP34, and *Trichoderma* sp. PBMP4 based on their morphological characteristics and multigene (ITS, *rpb2*, *tef-1*, *CaM*, and *BenA*) phylogenetic analyses. These four endophytic fungi could promote plant growth parameters under greenhouse conditions. Outstandingly, upland rice inoculated with *Tr. zelobreve* PBMP16 had a significant increase in total seed weight, root length, phenolic compounds, anthocyanin, antioxidants, and N uptake, which were higher than those of the noninoculated control, and even better than the chemical fertilizer. Overall, this report shows that endophytic fungi efficiently promoted growth and increased the phenolic compounds, anthocyanin, and antioxidants of Maled Phai rice.

## 1. Introduction

Rice (*Oryza sativa* L.) is the most popular staple food consumed worldwide, which is grown primarily in Asia. According to Chaudhari et al. [1], rice has a high nutritional value since it contains significant amounts of phytochemicals such as flavonoids, anthocyanins, carotenoids, phenolic compounds, and phytosterols [2]. In this work, the Maled Phai rice variety, which originated from Southern Thailand, was selected for investigation because this rice variety has been promoted as an economic crop across the country. The brown-purple seeds, a unique feature of Maled Phai rice, are rich in anthocyanin and other antioxidant compounds [3]. Interestingly, functional foods containing anthocyanins and phenolic compounds are now widely consumed due to their antioxidant activities beneficial to human health, especially in reducing the risk of coronary artery disease and cancer [4,5]. Unfortunately, due to increasing drought and scarcity of water in many agricultural areas, the growth and production of Maled Phai rice have become unsatisfactory, both in terms of yield and accumulation of beneficial bioactive compounds [6]. Currently, planting rice in upland areas is a promising agricultural management approach for crop production under water-limited conditions, without compromising the yield [7]. Finding appropriate techniques to promote upland rice management and increase its bioactive components is therefore important.

Plant growth-promoting methods include biological approaches such as the use of endophytic fungi, which are symbiotic inhabitants residing within leaves, stems, and roots of plants without causing harm to the hosts [8,9]. In this regard, endophytic fungi are known to increase plant growth through a variety of mechanisms, including the production of phytohormones, such as indole-3-acetic acid (IAA), gibberellic acid (GA_3_), cytokinins (Cks), etc. [10]. These hormones regulate plant growth and influence a wide range of plant developmental processes [11,12]. Endophytes also play a significant role in the solubilization of phosphate [13], the production of siderophores [14] and ammonia [15], increasing access to nutrients (N, P, K, Mg, etc.) [16], and also enhancing secondary metabolites (phenolic compounds, flavonoids, anthocyanin, etc.) in plants [17,18].

Several studies have reported the potential of endophytic fungi, which usually focus on plant growth promotion; however, their effects on the accumulation of phenolic compounds and anthocyanin, when analyzing both components simultaneously, in plants are still limited [19,20]. For example, the capability of endophytes to enhance phenolic compounds and anthocyanin production was examined with only a few types of plants, such as grapes and thale cress [18,21]. Therefore, this work aimed to investigate the plant growth-promoting properties of endophytic fungi isolated from upland black rice. The effects of endophytic fungi on an enhancement of phenolic compounds, anthocyanin, and their antioxidant activity in Maled Phai rice seeds under greenhouse conditions were also examined. The findings of this study could elucidate the potential to apply an eco-friendly method to help increase the accumulation of antioxidant compounds in rice seeds, which could thus add more value to Maled Phai rice and its products in the future.

## 2. Materials and Methods

### 2.1. Plant Sampling Site

Upland black rice was collected from Ban Haet District, Khon Kaen (16°9′57″ N, 102°48′12″ E), and Khao Kho District, Phetchabun (16°49′45.6″ N, 100°58′27.6″ E). Plant samples were randomly picked and taken to the laboratory for isolation and processing within 24 h of collection.

### 2.2. Isolation of Endophytic Fungi

The plant parts, including leaves, stems and roots, were washed with running tap water to remove adhered dust and debris, and then plant organs were cut into 1 × 1 cm segments. Successive surface sterilization was conducted according to the method of Yodsing et al. [22]. In short, plant segments were immersed in a series of solutions, as follows: 70% ethanol for 2 min, sodium hypochlorite solution for 5 min, and 70% ethanol for 30 s, followed by rinsing with sterile distilled water three times and drying using a sterile paper towel. In order to grow endophytic fungi, the segments of plant samples were transferred aseptically onto Petri plates containing half-strength potato dextrose agar (HPDA) (per liter: 100 g of potato, 10 g of dextrose, and 15 g of agar) supplemented with chlortetracycline (1000 mg·L^−1^) to avoid bacterial contamination. Then, the plates were incubated at room temperature under dark conditions. The growth of fungal colonies was observed every day for two weeks. The fungi growing out from the plant segments, which are considered endophytic fungi, were subsequently subcultured onto fresh PDA until a pure culture was obtained. Finally, the fungal endophytes were maintained on PDA slants at 4 °C for further experiments.

### 2.3. In Vitro Screening of Endophytic Fungi for Plant Growth-Promoting (PGP) Properties

#### 2.3.1. Phosphate Solubilization Assay

All endophytic fungal isolates were tested for their capacity to solubilize phosphate using Pikovskaya’s (PKV) broth (per liter: 0.5 g (NH_4_)_2_SO_4_, 0.1 g MgSO_4_⋅7H_2_O, 0.02 g NaCl, 0.02 g KCl, 0.003 g FeSO_4_⋅7H_2_O, 0.003 g MnSO_4_⋅H_2_O, 10.0 g glucose, 0.5 g yeast extract, and supplemented with 0.5% tricalcium phosphate) [23]. The endophytic fungal isolates were cultured for 7 days in 20 mL of PKV broth medium, and then incubated at 30 °C with shaking at 180 rpm. The control was made without fungal inoculation in the PKV medium. The culture was centrifuged at 4000 rpm for 10 min in order to collect the supernatant. Then, 2 mL of supernatant was mixed with 1 mL of 2.5% ascorbic acid, followed by 5 mL of 2% boric acid, 2 mL of Murphy’s reagent, and the final volume at 25 mL was made by adding 15 mL of deionized water. The solution mixture was incubated at room temperature for 30 min; thereafter, the absorbance was measured at a wavelength of 820 nm using a spectrophotometer (Hitachi High-Tech Science Corporation, Tokyo, Japan). The amount of soluble phosphate was calculated from the standard curve of the KH_2_PO_4_ standard solution [24].

#### 2.3.2. Indole-3-Acetic Acid (IAA) Production

The IAA production was determined from all isolates of endophytic fungi using a colorimetric technique performed with Salkowski’s method according to the method of Saengket et al. [25]. Firstly, the endophytic fungi were inoculated into a PDB medium supplemented with 0.2 mg·mL^−1^ of L-tryptophan. The inoculated broth was incubated at 30 °C, 180 rpm for 7 days. Noninoculated medium was set up as a control. After 7 days, the cultures were centrifuged at 4000 rpm for 10 min to retrieve the supernatant. Then, 1 mL of each sample solution was mixed with 2 mL of Salkowski reagent (2 mL of 0.5 M FeCl_3_ and 98 mL of 35% perchloric acid solution) and then incubated in the dark for 30 min. The color of the postreaction mixture developed a pink or red color, after which the optical density (OD) and absorbance at a wavelength of 530 nm were measured using a spectrophotometer. The concentration of IAA (μg·mL^−1^) of each potential fungal endophyte was compared to a standard curve of the standard IAA (Sigma-Aldrich, St. Louis, MO, USA).

#### 2.3.3. Gibberellic Acid (GA_3_) Production

The ability of the isolated endophytic fungi, in terms of GA_3_ production, was evaluated using a slightly modified method described by Syamsia et al. [26]. The endophytic fungi were cultured for 7 days in Czapek–Dox broth (CZ) and incubated at 30 °C with shaking at 180 rpm. The supernatant was collected via centrifugation at 4000 rpm for 10 min. A volume of 2 mL of zinc acetate reagent was added to 15 mL of culture supernatant, and the mixture was left undisturbed for 2 min. After that, 2 mL of potassium ferrocyanide was added and mixed well. The mixture was centrifuged at 4000 rpm for 10 min in order to collect the supernatant. Then, supernatant was mixed with an equal volume of 30% (*v*/*v*) HCl (approximately 5 mL) and the solution was incubated at 20 °C for 75 min. The GA_3_ quantification was assessed by measuring the absorbance at a wavelength of 254 nm using a spectrophotometer. The concentration of GA_3_ (μg·mL^−1^) of each potential fungal endophyte was compared to a standard curve of the standard GA_3_ (Solarbio, Beijing, China).

#### 2.3.4. Ammonia Production

All endophytic fungal isolates were tested for their capability to produce ammonia according to the method described by Mahfooz et al. [27], with some modifications. The endophytic fungal isolates were cultured for 7 days in peptone water and incubated at 30 °C with shaking at 180 rpm. The control was made with peptone water without fungal inoculation. The supernatant was collected via centrifugation at 4000 rpm for 10 min. Then, 1 mL of supernatant was added to 4 mL of Nessler’s reagent. The ammonia quantification was assessed by measuring the absorbance at a wavelength of 530 nm using a spectrophotometer. The progression from a brown to yellow color indicates a positive result for ammonia production. Ammonium sulfate solution ((NH_4_)_2_SO_4_) was used to produce a standard curve of ammonia [28] for the quantification of ammonium content in the samples. 

#### 2.3.5. Siderophore Production

Siderophore production was assessed by placing 0.5 cm of 1-week-old mycelia plugs onto Chrome Azurol S (CAS) agar containing hexadecyltrimethylammonium bromide (HDTMA), prepared by following the modified method of Schwyn and Neilands [29]. The noninoculated plate was used as a control. The plates were incubated at 28 °C for 5 days. Then, the appearance of an orange halo zone around the fungal colony was taken as indicative of siderophore production [30].

#### 2.3.6. Determination of Extracellular Enzyme Production

The four selected endophytic fungal isolates were investigated for their production of extracellular enzymes, including endoglucanase, pectinase, and xylanase, according to the method described by Kumla et al. [31]. An agar mycelia plug of the endophytic fungi was inoculated on tested agar media. Endoglucanase, pectinase, and xylanase productions were investigated on carboxymethyl cellulose (CMC), pectin agar, and xylan agar, respectively. The plates were incubated at 30 °C in darkness for 5 days. Endophytic fungal colonies were immersed in 1% Congo red for 30 min and then rinsed with 1 M NaCl for 15 min. A positive result was indicated when the colonies produced a yellow transparent circle that appeared around the colony. Four replications were made for each enzyme.

### 2.4. Identification of Endophytic Fungi

The endophytic fungi that exhibited the best plant growth-promoting properties were identified by their morphological characteristics and molecular identification.

#### 2.4.1. Morphological Identification

The identification of endophytic fungi is based on the morphological characteristics of the 7-day-old culture on the PDA plate. Colony and hyphae morphology, structures of the spore septa, and mycelium were visualized under a compound microscope (Nikon Eclipse 50i, Nikon Corporation, Tokyo, Japan) using a slide culture technique [32].

#### 2.4.2. Molecular Identification

Endophytic fungal isolates that exhibited plant growth-promoting properties were selected for identification. Genomic DNA from the fungal mycelia was extracted using 4% sodium dodecyl sulfate (SDS) following a modified method described by Niu et al. [33]. PCR amplification of the internal transcribed spacer (ITS) was amplified with primers ITS1/IT4 [11]. Partial sequences of RNA polymerase II second largest subunit (*rpb2*), translation elongation factor 1-alpha (*tef-1*), beta-tubulin (*BenA*), and calmodulin (*CaM*) gene regions were conducted with routine methods described by Nuangmek et al. [34], Zhang et al. [35], and Frisvad et al. [36]. The PCR products were observed via 1.5% agarose gel electrophoresis and then were purified using the GeneJETTM PCR purification kit (Fermentas, Toronto, Canada). Subsequently, the PCR products were sequenced at 1st Base Company (Kembangan, Malaysia). 

The sequences were analyzed by comparing them with the sequences in the GenBank database by BLASTn search. To reveal the phylogenetic position of selected fungal strains, the phylogenetic analyses of the representative taxa were conducted based on maximum likelihood (ML). Individual gene sequence datasets were aligned via MUSCLE [37]. ML analysis was carried out on RAxML-HPC2 version 8.2.10 [38] on the CIPRES web portal under the GTRCAT model, with 25 categories and 1000 bootstrap replications. The tree topologies were visualized in FigTree version 1.4.0.

### 2.5. Effect of Endophytic Fungi on the Growth Promotion of Maled Phai

#### 2.5.1. Endophytic Fungi Inoculum Preparation

Four fungal endophyte isolates possessing a wide range of plant growth-promoting properties, including the isolates PBMP16, PBMP28, KKMP34, and PBMP43, were prepared for an inoculum used for the next experiment. The selected fungal endophytes were inoculated onto PDA and incubated for 7 days. Then, agar plugs of fungal endophytes were transferred to a tube containing 50 g of sterilized sorghum seeds and incubated under static conditions until full colonization of the seeds was visible (approximately 2 weeks after incubation) [39]. 

#### 2.5.2. Preparation of Rice Seeds

Rice seeds were surface-sterilized by soaking in 6% sodium hypochlorite solution for 5 min, and then were rinsed with sterile distilled water for 3 min. Sterilized seeds were placed in a sterile Petri dish for 5 to 7 days to allow root germination. The seedlings were then grown in trays containing peat moss for 2 weeks. Thereafter, fully grown seedlings with a height of approximately 10 cm were selected for transplanting into plastic pots in the next experiment. 

#### 2.5.3. Experimental Design and Treatment

The pot experiment was carried out in the greenhouse at Khon Kaen University’s agronomy farm in Khon Kaen, Thailand. The experimental design was conducted by completely randomized design (CRD), and the fungal endophytes were set up for inoculation including the isolates PBMP16, PBMP28, KKMP34, and PBMP43. A noninoculated plant was used as a control. The treatment with chemical fertilizer application (15-15-15) at a ratio of 156.25 kg·ha^−1^ was arranged for a comparison with the inoculated treatments. All treatments were planted 1 plant per pot, and 6 replicates of each treatment were set up. The plastic pots were planted with endophytic fungi-inoculated seedlings, in which each of them contained sterilized sandy loam soil, 1 rice seedling, and 2 sorghum seeds infected with each endophytic fungal isolate. The physical chemicals of soil used in this experiment were as follows: pH 4.61, 0.71% organic matter, 0.033% total N, 19.50 mg of available P, 37.52 mg·kg^−1^ of available K, 44.39 mg·kg^−1^ of available Na, 273.79 mg·kg^−1^ of available Ca, and 34.14 mg·kg^−1^ of available Mg. The soil was sterilized by autoclaving twice at 121 °C for 1 h. The upland rice (Maled Phai) was grown in a greenhouse via irrigation using tap water for 90 days.

#### 2.5.4. Determination of Plant Growth Parameters

At 90 days after transplantation (DAT), plant height and the number of tillers were determined. The chlorophyll contents were analyzed according to the method described by Arnon [40]. Measurement of photosynthetic rate, stomatal conductance, and transpiration rate were determined from the second expanded leaves from the top of the main stem using a LI–6400XT portable photosynthesis system (LI–COR Bioscience, Lincoln, NE, USA). Water use efficiency (WUE) was calculated from the photosynthetic rate divided by the transpiration rate. At the harvest stage (120 DAT), the number of panicles and the weight of seeds were measured. Plant biomass was determined by measuring the dry weight of leaves, stems, and roots after drying in an oven at 80 °C for 3 days. Plant nutrient uptake concentration (N, P, K) was determined from shoot samples. The quality of plant roots including length, surface area, diameter, and volume were evaluated by scanning the root samples using an Epson scanner V800 PHOTO, and the data were analyzed using the WINRHIZO Pro2004 software (REGENT Instruments Inc., Quebec, QC, Canada). The colonization frequency (%CF) of endophytic fungi was determined according to the method described by Mehmood et al. [41]. In short, plant roots were collected at the harvest stage. Then, they were initially washed with tap water, and cut into small pieces. The root samples were then heated at 90 °C in a 2.5% KOH solution for 5 min followed by treatment with 1% HCl for 5 min, then stained with acetic glycerine solution (trypan blue 1 g, glycerine 500 mL, 1% hydrochloric 50 mL, distilled water 450 mL) and destained in lactophenol solution. Root segments (30 segments) were visualized under a compound microscope (Nikon Eclipse 50i, Nikon Corporation, Tokyo, Japan), and the percentage of root colonization was measured by dividing the number of root segments positive for colonization by the total number of observed root segments and multiplying by 100%.

#### 2.5.5. Determination of Functional Substances

The extraction procedure for anthocyanin, phenolic compounds, and the 1,1-diphenyl-2-picrylhydrazyl radical (DPPH) scavenging assay for antioxidant properties were conducted according to the method described by Kapcum et al. [4], with some modifications. Briefly, dried seeds (1.0 g) were subjected to phenolic compound extraction using 10 mL of methanol, and the mixture was then vigorously shaken for 2 h. The methanol layer was separated from the seed fraction via centrifugation at 3000 rpm for 10 min. The liquid was filtered through a paper filter Whatman No. 1. The seed residues were re-extracted twice with 5 mL of methanol following the same procedure. Three aliquots obtained from different rounds of extraction were combined and then stored at −18 °C in the dark until further analysis. 

The content of phenolic compounds in the seed extracts was determined using the Folin–Ciocalteu method. A volume of 125 µL of extracts was mixed with 250 µL of Folin–Ciocalteu reagent and 3 mL of distilled water. The mixture was allowed to stand for 6 min and then was added to 2.5 mL of 7% sodium carbonate solution. The mixture was incubated at room temperature for 90 min prior to absorbance measurement at a wavelength of 760 nm using a spectrophotometer (Hitachi High-Tech Science Corporation, Tokyo, Japan). The absorbance values were compared to the standard curve of gallic acid for quantification of phenolic compound content. The results were expressed as mg of gallic acid equivalent per 100 g sample. 

The anthocyanin content was determined using the pH differential spectrophotometric method. Briefly, 50 µL of the seed extract was separated into two aliquots. One of them was added to 3 mL of 0.025 M potassium chloride buffer (pH 1.0), and the other with 3 mL of 0.4 M sodium acetate buffer (pH 4.5). The mixture was then allowed to stand for 20 min before measuring absorbance at 520 and 700 nm. Total anthocyanin content was expressed as cyanidin-3-glucoside (Cy-3-G) equivalents per 100 g sample according to the following formula:

(1) $$\mathrm{Total}\;\mathrm{anthocyanin}\;(\mathrm{mg}\cdot 100\;g^{-1})=\frac{∆A\times \mathrm{MW}\times DF\times (V/D)\times 100}{\epsilon \times L}$$

where ∆A = (A520 nm–A700 nm) pH 1.0–(A520 nm–A700 nm) pH 4.5, ϵ—molar extinction coefficient of Cy-3-G (29,600 M^−1^ cm^−1^), L—path length of the cuvette (1 cm), MW—molecular weight of anthocyanins (449.2 g·mol^−1^), DF—dilution factor, V—final volume (mL), and G—the weight of the sample (g).

DPPH free radical scavenging activity was determined using the method of Leong and Shui, with modifications [42]. An aliquot of 100 µL sample (with appropriate dilution) was mixed with 4.0 mL of 0.05 mM DPPH solution. The mixture was then incubated for 30 min at room temperature prior to absorbance measurement at a wavelength of 517 nm using a spectrophotometer (Hitachi High-Tech Science Corporation, Tokyo, Japan). The percentage of radical scavenging ability was calculated using the following formula:

(2) $$\%\mathrm{DPPH}\;\mathrm{scavenging}=\frac{A_{0}-A_{1}}{A_{0}}\;\times \;100$$

where A_0_ is the absorbance at 515 nm of the control, and A_1_ is the absorbance at 517 nm of the sample.

### 2.6. Statistical Analysis

Analysis of variance (ANOVA) was conducted using the Statistix 10 software. Data were analyzed according to a completely randomized design (CRD) from six plot replications. The least significant difference (LSD) test was applied to test the significant difference among the treatment means at *p* ≤ 0.05.

## 3. Results

### 3.1. Isolation of Endophytic Fungi

A total of 56 endophytic fungi were isolated from leaves, shoots, and root tissues of Thai pigmented-upland rice varieties, Dam Hmong and Maled Phai, collected from rice fields in Khon Kaen and Phetchabun Provinces in Thailand. All fungal endophytes were tested for their ability to produce plant growth-promoting properties.

### 3.2. In Vitro Screening of Endophytic Fungi for Plant Growth-Promoting (PGP) Properties

All endophytic fungal isolates were evaluated for PGP properties including phosphate solubilization activity, IAA production, GA_3_ production, ammonia production, and siderophore production, as shown in Table 1. The results revealed that all fungal isolates could solubilize phosphate. The highest phosphate solubilization activity was observed for isolate PBMP43 at 2325.83 μg·mL^−1^, followed by KKMP10 (1692.50 μg·mL^−1^) and KKDM04 (1350.00 μg·mL^−1^). In terms of their ability to produce phytohormones (IAA, GA_3_), the highest concentration of IAA was found in isolate KKDM04, which produced an amount of IAA of 318.48 μg·mL^−1^, followed by isolates PBMP43 (314.17 μg·mL^−1^) and PBMP16 (307.17 μg·mL^−1^), all of which were significantly different from that of other isolates. In addition, the highest GA_3_ production was found in the isolate PBMP43 at the value of 301.35 μg·mL^−1^, followed by 298.46 μg·mL^−1^ and 241.34 μg·mL^−1^, which were found in the isolates PBMP16 and KKMP34, respectively. These isolates produced a considerable amount of ammonia. The results showed that isolate PBMP43 produced the highest ammonia concentration of 65.69 μg·mL^−1^, followed by the isolates PBMP16 (51.87 μg·mL^−1^) and KKMP34 (45.23 μg·mL^−1^). According to the ability of endophytic fungi to produce siderophores, the results revealed that endophytic fungal isolates PBMP28 and PBMP34 exhibited a color change on CAS agar from blue to orange, indicating that these isolates could produce siderophores. Therefore, these four isolates, PBMP16, PBMP28, KKMP34, and PBMP43, were selected for investigation with plants in pots under greenhouse conditions due to their efficient plant growth-promoting properties. 

The ability of endophytic fungi to produce extracellular enzymes including endoglucanase, pectinase, and xylanase is shown in Table 1. The results revealed that endophytic fungal isolates PBMP16 and PBMP43 produced endoglucanase and pectinase, while isolates PBMP28 and PBMP34 were able to produce only one enzyme (pectinase). However, xylanase production was not observed in any of the endophytic fungal isolates. 

### 3.3. Identification of the Selected Endophytic Fungal Isolates

**Figure 1:**
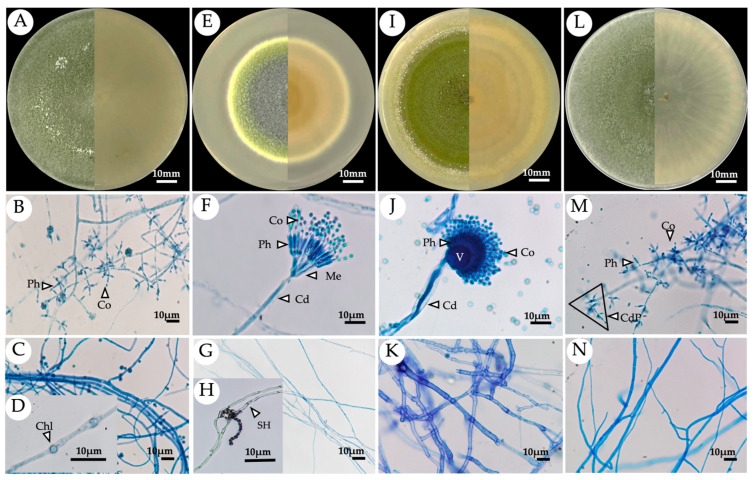
Morphological observation of the respective fungal endophytes under a light microscope. (**A**–**D**) *Trichoderma* sp. PBMP16; (**E**–**H**) *Talaromyces* sp. PBMP28; (**I**–**K**) *Aspergillus* sp. KKMP34; and (**L**–**N**) *Trichoderma* sp. PBMP43. Phialide (Ph), conidia (Co), conidiophore (Cd), conidiophore pyramidal (CdP), chlamydospore (Chl), metula (Me), vesicle (V), and septate hyphae (SH).

The four selected endophytic fungal isolates were initially identified by their morphological characteristics. Pure culture of each isolate was grown on PDA medium for 7 days, and then their microscopic characteristics were observed under a light microscope. It was found that the morphologies of these isolates (Figure 1) were most similar to those of the members of three different genera including *Aspergillus*, *Talaromyces*, and *Trichoderma.* However, the identification of these four selected endophytic fungal isolates was subsequently confirmed by multigene phylogenetic analyses. GenBank accession numbers of the sequences obtained in this study are shown in Table 2.

**Figure 2:**
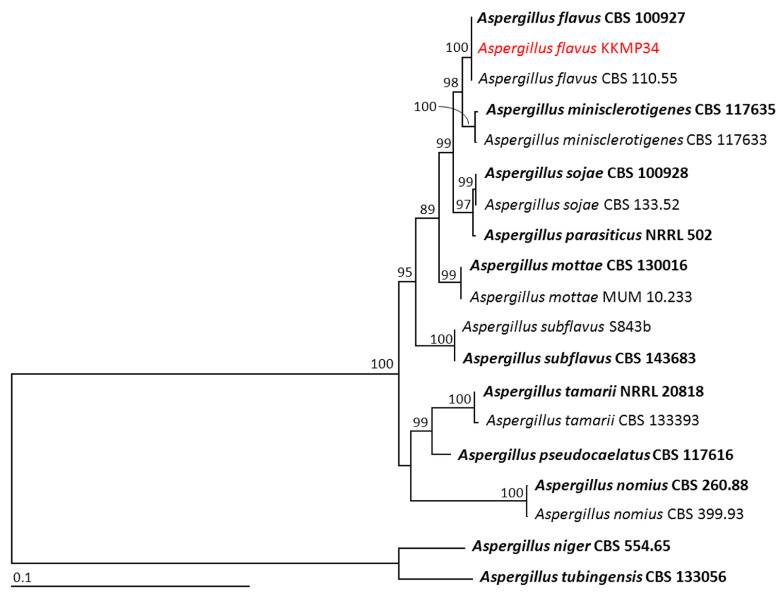
Phylogram derived from maximum likelihood analysis of 19 fungal isolates of the combined ITS, *BenA*, and *CaM* genes. *Aspergillus niger* CBS 554.65 and *A. tubingensis* CBS 133056 were set as the outgroup. The numbers above branches represent bootstrap percentages, and values > 75% are shown. The scale bar represents the expected number of nucleotide substitutions per site. The fungal isolate obtained from this study is in red. Type species are in bold.

Phylogenetic analysis of genera *Aspergillus* and *Talaromyces* were based on ITS, *BenA*, and *CaM* sequences, while ITS, *rpb2*, and *tef-1* sequences were used for the genus *Trichroderma*. For phylogenetic analysis of genus *Aspergillus*, the aligned matrix of the combined gene dataset consisted of 1612 characters, including gaps (1–555 bp for ITS, 556–1028 bp for *BenA*, and 1029–1612 bp for *CaM*). *Aspergillus niger* CBS 554.65 and *A. tubingensis* CBS 133056 were set as the outgroup. The sequence alignment has 307 distinct alignment patterns, with 7.63% of undetermined characters or gaps. The RAxML analysis resulted in a final ML optimization likelihood value of –4976.8049. The phylogenetic tree is shown in Figure 2. The results indicated *Aspergillus* sp. KKMP34 clumped together with *A. flavus*, which included the type species CBS 100927. This clade established a monophyletic clade with 100% BS support. Therefore, *Aspergillus* sp. KKMP34 was identified as *A. flavus.*

**Figure 3:**
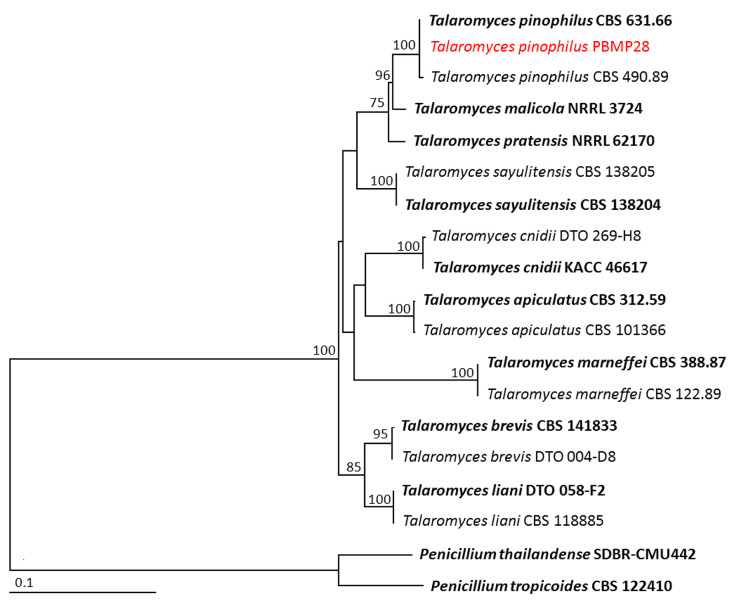
Phylogram derived from maximum likelihood analysis of 19 fungal isolates of the combined ITS, *BenA*, and *CaM* genes. *Penicillium thailandense* SDBR-CMU442 and *P. tropicoides* CBS 122410 were set as the outgroup. The numbers above branches represent bootstrap percentages, and values > 75% are shown. The scale bar represents the expected number of nucleotide substitutions per site. The fungal isolate obtained from this study is in red. Type species are in bold.

For phylogenetic analysis of genus *Talaromyces*, the aligned matrix of the combined gene dataset consisted of 1414, characters including gaps (1–515 bp for ITS, 516–901 bp for *BenA*, and 902–1414 bp for *CaM*). *Penicillium thailandense* SDBR-CMU442 and *P. tropicoides* CBS 122410 were set as the outgroup. The sequence alignment has 360 distinct alignment patterns, with 11.55% of undetermined characters or gaps. The RAxML analysis resulted in a final ML optimization likelihood value of –5335.3472. The phylogenetic tree successfully assigned *Talaromyces* isolate PBMP28 within the same clade of *T*. *pinophilus*, which consisted of the type species CBS 631.66 (Figure 3). This clade established a monophyletic clade with 100% BS support. Therefore, *Talaromyces* isolate PBMP28 was identified as *T*. *pinophilus*.

**Figure 4:**
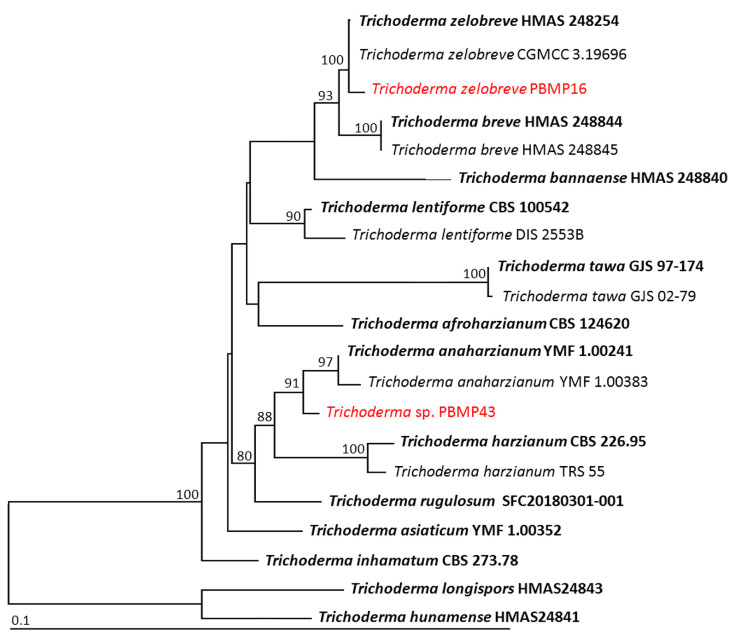
Phylogram derived from maximum likelihood analysis of 21 fungal isolates of the combined ITS, *rpb2*, and *tef-1*. *Trichroderma longispors* HMAS24843 and *T. hunamense* HMAS24841 were set as the outgroup. The numbers above branches represent bootstrap percentages, and values > 75% are shown. The scale bar represents the expected number of nucleotide substitutions per site. The fungal isolates obtained from this study are in red. Type species are in bold.

The combined sequences of ITS, *rpb2*, and *tef*-1 were utilized for the phylogenetic analysis of genus *Trichoderma*. The aligned matrix of the combined gene dataset consisted of 1991 characters including gaps (1–580 bp for ITS, 581–1564 bp for *rpb2*, and 1565–1991 bp for *tef*-1). *Trichroderma longispors* HMAS24843 and *T*. *hunamense* HMAS24841 were set as outgroups. Sequence alignment has 408 distinct alignment patterns, with 10.11% of undetermined characters or gaps. The RAxML analysis resulted in a final ML optimization likelihood value of –6277.5993. The phylogenetic tree is shown in Figure 4. *Trichoderma* isolate PBMP16 was successfully assigned within the same clade of *Tr. zelobreve*, which consisted of the type species HMAS 248254. Therefore, PBMP28 was identified as *Tr. zelobreve*. Furthermore, it was found that *Trichoderma* isolated PBMP43 was distinguished from the previously known species of *Trichoderma*. This isolation was closely related to *Tr. anaharzianum* and *Tr. harzianum*. However, both *rpb2* and *tef-1* sequences showed similarity values less than 97.5% to type species of *Tr. anaharzianum* and *Tr. harzianum*. Therefore, this isolate was categorized as a previously unrecognized species, and further study would be required for its identification.

### 3.4. Effect of Endophytic Fungi on the Growth Promotion of Upland Rice in Pot under Greenhouse Conditions

**Figure 5:**
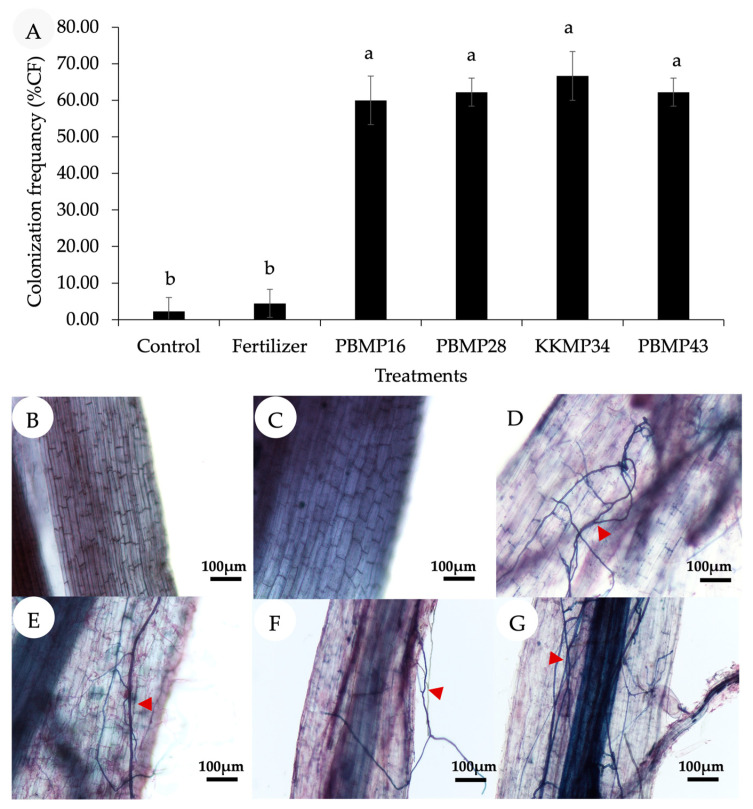
The colonization frequency of endophytic fungi in the root of Maled Phai at the harvest stage (120 DAT) in pot trials under greenhouse conditions. (**A**) Colonization frequency (%); (**B**) control; (**C**) chemical fertilizer application; (**D**) inoculation with *Tr. zelobreve* PBMP16; (**E**) inoculation with *T. pinophilus* PBMP28; (**F**) inoculation with *A. flavus* KKMP34; and (**G**) inoculation with *Trichoderma* sp. PBMP43. The hypha of fungal endophytes colonizing in the root of upland black rice are represented by red arrows. Different letters on the bar indicate significant differences of the mean values (*p* ≤ 0.01 using the LSD test).

In order to investigate the effect of endophytic fungi on growth promotion of Maled Phai rice, the plant growth parameters at 90 DAT were determined, as shown in Table 3. The results showed that all plant growth parameters of the fungi-inoculated plants were improved, including plant height, number of tillers, total chlorophyll content, and photosynthesis rate, which were significantly higher than those of the noninoculated plants. In particular, plant height, chlorophyll content, photosynthesis, and stomatal conductance qualities of the *Tr. zelobreve* PBMP16-inoculated plants were even higher than those of the plants administered chemical fertilizer. Root colonization in Maled Phai rice by the endophytic fungi was measured at 120 DAT (the harvest stage). The results in Figure 5 showed that all fungi-inoculated plants had significantly higher colonization than that of noninoculated plants. In particular, *A. flavus* KKMP34 showed the highest percentage of root colonization at 66.67%. These results confirmed that all endophytic fungi played an important role in promoting the growth of rice. Notably, the endophytic fungi colonization in the roots of chemical fertilizer-treated plants was not significantly different from those in noninoculated plants, which was much lower than the endophytic fungi-inoculated plants. This suggested that chemical fertilizer had no benefit on the enhancement of root colonization by indigenous endophytes. Thus, an additional inoculation of the effective plant growth-promoting endophytes is the most important factor.

At the harvest stage, panicle number, total seed weight, shoot weight, root fresh weight, and root dry weight of Maled Phai rice were determined, as shown in Table 4. The results indicated that all plant growth parameters of the inoculated plants significantly increased when compared to the noninoculated plants (control). Although plants treated with chemical fertilizer had a higher panicle number, total shoot weight, and root fresh and dry weight than the other treatments, the inoculation of *Tr. zelobreve* PBMP16 provided favorable total shoot weight and root fresh weight of the plants at a level comparable to the effects of chemical fertilizer. Moreover, the total seed weight of Maled Phai rice was the highest in plants inoculated with *Tr. zelobreve* PBMP16, which was even higher than the chemical fertilizer-treated plants. This suggests that all endophytic fungi in this study efficiently enhanced the growth and yield of Maled Phai rice, especially *Tr. zelobreve* PBMP16, which even outperformed the chemical fertilizer.

Table 5 presents the quality of plant root growth in terms of length, surface area, diameter, and volume of Maled Phai roots at the harvest stage. The results revealed that all treatments with endophytic fungi showed plant root quality higher than those of the noninoculated control. In particular, plants inoculated with *Trichoderma* sp. PBMP43 showed the best root length, surface area, diameter, and root volume, which were comparable to those of plants treated with chemical fertilizer. Furthermore, *Tr. zelobreve* PBMP16 also had a profound enhancement effect on root length, diameter, and volume, which were comparable to those of plants treated with *Trichoderma* sp. PBMP43, and even higher than the chemical fertilizer-treated plants in some cases. All of these results suggest that endophytic fungi provided better root quality than the noninoculated control, in which *Tr. zelobreve* PBMP16 and *Trichoderma* sp. PBMP43 offered the most noticeable effects.

The determination of phenolic compounds, anthocyanin, and antioxidant activity of rice seeds harvested from plants grown with the endophytic fungi is shown in Table 6. The results indicated that most plants inoculated with endophytic fungi exhibited higher TPC, TAC, and antioxidants than those of the uninoculated plants and the plants treated with chemical fertilizer. Remarkably, rice seeds of plants inoculated with *Tr. zelobreve* PBMP16 showed the highest concentrations of phenolic compounds, anthocyanin, and antioxidants (% radical scavenging). Additionally, plants inoculated with endophytic fungi had a significant increase in nitrogen, phosphorus, and potassium uptake, which were higher than the noninoculated control. This demonstrated that endophytic fungi could effectively facilitate nutrient uptake in Maled Phai rice, and thus enhance the accumulation of bioactive compounds in rice seeds.

The correlations between endophytic fungi and % colonization with plant parameters of rice at the harvest stage are presented in Table S1. The endophytic fungal colonization rate had a negative correlation with the number of panicles and P uptake. In contrast, it had a positive correlation with plant height, number of tillers, total chlorophyll, Pn, Sc, Tr, WUE, total seed weight, total shoot weight, root FW, root DW, TAC, TPC, antioxidant, and N and K uptake. These results suggest that endophytic fungi were the dominant factor affecting plant growth performance and phytochemical (total phenolic compound and anthocyanin) content.

## 4. Discussion

Generally, endophytic fungi are beneficially used in agriculture since they can promote the growth and yield of many plants, and are considered as one of the main factors affecting plant bioactive compounds and secondary metabolites [43]. In this study, fifty-six endophytic isolates isolated from *Oryza sativa* L. were investigated for their plant growth-promoting properties, including phosphate solubilization, IAA and GA_3_ production, ammonia production, and siderophore production. We found that four fungal isolates, including PBMP16, PBMP28, KKMP34, and PBMP43, could effectively produce PGP substances. They were then identified to species level on the basis of morphological characteristics and an extensive multilocus (ITS, *rpb2*, *tef-1*, *CaM*, and *BenA* genes) phylogenetic analysis. These isolates were identified as *Trichoderma zelobreve* PBMP16, *Talaromyces pinophilus* PBMP28, *Aspergillus flavus* KKMP34, and *Trichoderma* sp. PBMP43, respectively. These endophytic fungal species were commonly found in rice, similar to a previous study showing the presence of endophytic fungal isolates *Aspergillus* sp., *Penicillium* sp., and *Trichoderma* sp. In rice [44].

Phosphate solubilization was determined because phosphorus (P) is one of the vital nutrients for overall plant growth and productivity [45]. In this study, the highest level of P solubilization was found in *Trichoderma* sp. PBMP43 (2325.83 μg·mL^−1^) and *Tr. Zelobreve* PBMP16 (1176.67 μg·mL^−1^), which was much higher than that of other previous reports. For example, Radhakrishnan et al. [46] showed that the phosphate solubilization ability of *Fusarium verticillioides* RK01 was in a range of 261.9 ± 1.6 μg·mL^−1^. Moreover, several species of *Penicillium* spp. Showed relatively lower levels of phosphate solubilizing activity such as 220 μg·mL^−1^ by *P. oxallicum*, 230 μg·mL^–1^ by *P. citrinum*, and 210 μg·mL^–1^ by *P. purpurogenum* [47]. This confirmed that the P solubilization activity of *Trichoderma* sp. PBMP43 and *Tr. Zelobreve* PBMP16 exerted such action on many other endophytes.

One of the most important plant growth-promoting abilities of endophytic fungi is the production of phytohormones, including auxin [such as indole-3-acetic acid (IAA)] and gibberellins [also known as, gibberellic acid (GA_3_)], which are important substances for plant developmental processes [16,48]. In our study, the highest amount of IAA of 314.56 μg·mL^−1^ was produced by the endophytic fungus *Trichoderma* sp. PBMP43, followed by 307.17 μg·mL^−1^ produced by *Tr. zelobreve* PBMP16. Such an amount was obviously higher than that of the other reported members in this species. For example, the *T. harzianum* strain FCCT 363-2v was able to produce an IAA of 21.14 ± 0.003 μg·mL^−1^ [49], and the *T. harzianum* strain InaCC F88 produced an IAA of 9.22 μg·mL^−1^ [50]. Furthermore, *Trichoderma* sp. PBMP43 and *Tr. zelobreve* PBMP16 showed a strong production of GA_3_ of 301.35 and 298.46 μg·mL^−1^, respectively, which was also much higher than that of previous reports. For example, Nath et al. [51] demonstrated that *F. oxysporum* was able to produce a GA_3_ of 12.46 ± 0.84 μg·mL^−1^, and *P. chrysogenum* F1 produced a GA_3_ of 10.95 ± 0.37 μg·mL^−1^. This suggests that our endophytic fungi have great potential to promote the growth and yield of rice.

Furthermore, our results indicated that the endophytic fungi *Trichoderma* sp. PBMP43 and *Tr. zelobreve* PBMP16 exhibited the best efficiency in ammonia production among other isolates. Due to ammonia being a good source of nitrogen for plants, these isolates then have the potential to provide additional nitrogen sources for the development of plant biomass, increased photosynthesis rate, and possible enhanced yield [52].

Although *Trichoderma* sp. PBMP43 and *Tr. zelobreve* PBMP16 outperformed other isolates in producing several PGP substances, the production of siderophores was, in contrast, found the highest in *T. pinophilus* PBMP28 and *A. flavus* KKMP34. Since siderophores are a key component regulating iron acquisition by plant pathogens [53], these isolates are likely to benefit plant growth in terms of being a biocontrol agent.

Another important characteristic of plant growth promoters is the ability of endophytic fungi to produce extracellular enzymes such as endoglucanase, pectinase, and xylanase. These enzymes might facilitate endophytes to colonize plant tissue faster through the hydrolysis of plant cell walls, allowing the plants to absorb more water and nutrients [11]. Another mechanism through which microorganisms help to inhibit pathogenic organisms in plants is the production of cell wall-degrading enzymes of pathogens, especially cellulase (endoglucanase, exoglucanase, and *β*-glucosidase) [54,55]. In our study, the ability of four endophytic fungi to produce different types of enzymes was dependent upon species and strain, which is similar to previous reports of Hawar et al. [56], who reported that endophytic fungi isolated from *Ziziphus spina* have the ability to produce different extracellular enzymes. This indicates that the enzyme production differs between fungi, and often corresponds to the requirements of its habitat. This may be due to many factors changing in the host related to age and environmental factors [57]. Notably, *Trichoderma* sp. PBMP43 and *Tr. zelobreve* PBMP16 produced both enzymes endoglucanase and pectinase. This suggests these two strains might be able to promote plant growth better than the other strains.

In this research, four isolates of endophytic fungi, including *Tr. zelobreve* PBMP16, *T. pinophilus* PBMP28, *A. flavus* KKMP34, and *Trichoderma* sp. PBMP43, were selected for use as inocula in pot experiments because they exhibited desirable PGP properties, while possibly having different mechanisms. The results of this study showed that all endophytic fungi promoted Maled Phai rice higher than those of the noninoculated control in all parameters. Particularly, *Trichoderma* sp. PBMP43 and *Tr. zelobreve* PBMP16 promoted better plant growth than *T. pinophilus* PBMP28 and *A. flavus* KKMP34. Therefore, PGP substance properties are more important than the efficient production of siderophores. These results are consistent with Mohamed et al. [58], who showed that *P. chrysogenum* NGB-WFS2, which could highly solubilize phosphorus and IAA production, while having a low percentage of siderophore production, could promote plant growth better than *A. versicolor* NGB-WFS9, which could produce a high amount of siderophores. However, it was found that chemical fertilizers were more effective in promoting some characteristics of Maled Phai rice than endophytes. This result was similar to the findings of Khaekhum et al. [59], suggesting that plants treated with chemical fertilizer could promote plant height, stem diameter, and SPAD values greater than the fungus *Exserohilum rostratum* NMS1.5. This might be due to chemical fertilizers providing nutrients that are ready to use for plants, while endophytic fungal inoculation sometimes needs sufficient root colonization to affect plant growth. 

Interestingly, plants inoculated with *Trichoderma* sp. PBMP43 and *Tr. zelobreve* PBMP16 also increased the accumulation of the functional substances (total phenolic compounds, anthocyanin content, and antioxidant activity) in Maled Phai rice seeds more than those of the plants treated with chemical fertilizer. This suggests that chemical fertilizer tended to supply nutrients mainly to accelerate the growth of plant physical characteristics, leaving insufficient nutrients to promote the accumulation of functional substances. In contrast, our endophytic fungi enhanced plant growth and accumulation of functional substances. The benefits of using *Trichoderma* have been reported quite frequently for increasing the active ingredients and giving positive results. For instance, Velasco et al. [60] reported cabbage and turnip greens inoculated with *T. hamatum* had a significant increase in their phytochemical content, such as glucosinolates and antioxidant compounds. Lombardi et al. [61] showed that *Trichoderma* sp. strains T22, TH1, and GV41 inoculated on strawberry significantly increased the total content of anthocyanins (31 and 66% for GV41 and TH1, respectively), ascorbic acid (23% for GV41), and corresponding antioxidant capacity (8% for GV41). Ortega-Garcia et al. [62] reported that secondary metabolites in onions were increased when cocultured with *Trichoderma*, but depended on plant genotype.

Our study suggests that using endophytes instead of chemical fertilizer is preferable because the Maled Phai rice strain is commonly planted for its functional properties, which lend themselves to the production of medicines, food supplements, and other value-added rice products. Furthermore, the presence of high contents of antioxidant compounds in purple rice seeds results in an increased market price, especially when the rice is organically produced without chemicals applied during cultivation [63]. This present work proved that the use of endophytes, particularly *Trichoderma* sp. PBMP43 and *Tr. zelobreve* PBMP16, effectively increases plant growth and the accumulation of functional substances better than the use of chemical fertilizer. Although the use of endophytic fungi alone had substantial benefits to plant growth and yield, as mentioned earlier, the use of chemical fertilizers tended to promote some physical plant characteristics faster than the use of endophytes. Therefore, in terms of agricultural management practices, it may be necessary to consider the use of chemical fertilizers in combination with biological methods to obtain the highest efficiency (i.e., cost-effectiveness, fast growth, and high yields while maintaining environmentally friendly practices). In this regard, the inoculation of our endophytic fungi could help farmers reduce the production cost for Maled Phai rice by replacing chemical fertilizer at the partial dosage or even full dosage with proper management, whereas the quality of functional substances in rice seeds could be improved to meet market demand. The sustainable use and management of fertilizers in conjunction with endophytic fungal inoculation is the main focus for further investigation in the field trials of our research group.

## 5. Conclusions

Out of 56 fungal endophytes isolated from upland black rice, 4 isolates, including *Tr. zelobreve* PBMP16, *T. pinophilus* PBMP28, *A. flavus* KKMP34, and *Trichoderma* sp. PBMP43, were the most effective for phosphate solubilization, and IAA, GA_3_, ammonia, and siderophore production. They had the ability to increase several plant growth parameters such as height, number of tillers, total chlorophyll, photosynthesis rate, panicle number, total seed weight, total shoot weight, root weight, and plant root growth in pot trials under greenhouse conditions. These endophytic fungi also enhanced phytochemical (total phenolic compounds and anthocyanin) accumulation and antioxidants in rice seeds. Remarkably, an endophytic fungus, *Tr. zelobreve* PBMP16, had the best performance in promoting plant growth while also enhancing phytochemicals in Maled Phai rice seeds even more than the use of chemical fertilizer, in which previous studies analyzing the simultaneous accumulation of phenolic compounds and anthocyanins in plants using endophytic fungi were limited. Note that this study shows the successful application of endophytic fungi for growth promotion and increased functional compounds in Maled Phai rice seeds. These results strongly support the potential use of endophytic fungi as an alternative method to promote the quality of rice in place of chemical fertilizer.

## Figures and Tables

**Table 1:** Plant growth-promoting properties of endophytic fungi isolated from upland black rice.

Isolates	Phosphate Solubilization (μg·mL^−1^)	IAA Production (μg·mL^−1^)	GA_3_ Production (μg·mL^−1^)	Ammonia Production (μg·mL^−1^)	Siderophore Production	Extracellular Enzyme Production		
						Endoglucanase	Pectinase	Xylanase
PBMP16	1176.67 ^de^	307.17 ^a^	298.46 ^a^	51.87 ^b^	−	+	+	−
KKDM22	897.31 ^f^	266.90 ^b^	89.23 ^g^	35.41 ^d^	−	nd	nd	nd
PBMP28	1082.53 ^e^	273.29 ^b^	162.86 ^d^	44.27 ^c^	+	+	−	−
KKDM04	1350.00 ^c^	318.48 ^a^**	23.64 ^i^	12.45 ^g^	+	nd	nd	nd
KKMP34	1055.83 ^ef^	160.84 ^d^	241.34 ^b^	45.23 ^c^	+	−	+	−
KKMP28	1470.14 ^c^	235.49 ^c^	132.76 ^e^	32.71 ^d^	−	nd	nd	nd
KKMP10	1692.50 ^b^	111.73 ^e^	76.21 ^h^	18.50 ^f^	−	nd	nd	nd
PBMP43	2325.83 ^a^**	314.17 ^a^	301.35 ^a^**	65.69 ^a^**	−	+	+	−
KKMP27	1336.67 ^cd^	217.77 ^c^	106.97 ^f^	27.97 ^e^	−	nd	nd	nd
KKDM45	1085.00 ^e^	109.67 ^e^	212.50 ^c^	32.54 ^d^	−	nd	nd	nd

Different letters indicate significant differences among values within the same column using the LSD test. **, Significantly different (*p* ≤ 0.01 using the LSD test). “+”: Positive result; “−”: negative result; nd: not determined.

**Table 2:** GenBank accession number of EPF isolated from Thai pigmented-upland rice.

Endophytic Fungi Species	Isolate	GenBank Accession Number				
		ITS	*CaM*	*BenA*	*rpb2*	*tef-1*
*Aspergillus flavus*	KKMP34	LC738615	OR488618	OR488616	–	–
*Talaromyces pinophilus*	PBMP28	LC738614	OR488619	OR488617	–	–
*Trichoderma* *zelobreve*	PBMP16	LC738613	–	–	OR488620	OR488622
*Trichoderma* sp.	PBMP43	LC738616	–	–	OR488621	OR488623

**Table 3:** Plant height, tiller number, total chlorophyll, photosynthesis rate (Pn), transpiration rate (Tr), stomatal conductance (Sc), and water use efficiency (WUE) of the inoculated plants compared to those of the noninoculated plants at 90 DAT.

Treatments	Height (cm)	Number of Tillers	Total Chlorophyll (mg·L^−1^)	Pn (μmol CO_2_·m^−2^·s^−1^)	Tr (mmol H_2_O·m^−2^·s^−1^)	Sc (mmol H_2_O·m^−2^·s^−1^)	WUE (μmol CO_2_ mmol·H_2_O^−1^)
Control	85.00 ^e^	4.50 ^c^	29.83 ^e^	16.55 ^d^	3.08 ^b^	0.25 ^b^	5.37
Fertilizer	86.33 ^d^	15.50 ^a^	39.23 ^d^	17.30 ^d^	3.77 ^ab^	0.31 ^b^	4.59
*A. flavus* KKMP34	94.42 ^bc^	7.50 ^b^	38.25 ^d^	21.10 ^b^	3.77 ^ab^	0.29 ^b^	5.62
*T. pinophilus* PBMP28	93.00 ^c^	7.00 ^b^	41.98 ^c^	19.97 ^bc^	3.60 ^b^	0.28 ^b^	5.59
*Tr. zelobreve* PBMP16	97.00 ^a^	7.33 ^b^	48.59 ^b^	22.35 ^a^	4.42 ^a^	0.44 ^a^	5.15
*Trichoderma* sp. PBMP43	95.40 ^ab^	7.83 ^b^	50.47 ^a^	19.70 ^c^	3.56 ^b^	0.29 ^b^	5.58
F-test	**	**	**	**	*	*	ns
%CV	5.12	13.77	17.16	11.08	14.49	20.92	11.61

Different letters indicate significant differences among values within the same column using the LSD test. *, Significant difference at *p* ≤ 0.05; **, significant difference at *p* ≤ 0.01; ns, nonsignificant difference. Pn, photosynthesis rate; Tr, transpiration rate; Sc, stomatal conductance; WUE, water use efficiency.

**Table 4:** The number of panicles, total seed weight, total shoot weight, and root weight of the inoculated plants compared to those of the noninoculated plants at the harvest stage.

Treatments	Number of Panicles	Total Seed Weight (g·Plant^−1^)	Total Shoot Weight (g·Plant^−1^)	Root (g·Plant^−1^)	
				Fresh Weight	Dry Weight
Control	3.83 ^d^	3.59 ^c^	15.69 ^d^	102.57 ^c^	11.78 ^d^
Fertilizer	9.17 ^a^	8.85 ^b^	36.16 ^a^	131.08 ^a^	28.23 ^a^
*A. flavus* KKMP34	5.83 ^bc^	8.77 ^b^	27.15 ^c^	121.96 ^ab^	20.67 ^bc^
*T. pinophilus* PBMP28	5.00 ^cd^	7.75 ^b^	26.53 ^c^	114.29 ^bc^	17.55 ^c^
*Tr. zelobreve* PBMP16	6.50 ^b^	10.69 ^a^	32.66 ^ab^	123.14 ^ab^	20.84 ^bc^
*Trichoderma* sp. PBMP43	6.50 ^b^	8.79 ^b^	28.58 ^bc^	123.55 ^ab^	21.12 ^b^
F-test	**	**	**	*	**
%CV	30.49	30.53	26.83	12.43	27.45

Different letters indicate significant differences among values within the same column using the LSD test. *, Significant difference at *p* ≤ 0.05; **, significant difference at *p* ≤ 0.01.

**Table 5:** Plant root growth parameters including length, surface area, diameter, and volume of Maled Phai rice at the harvest stage under pot conditions.

Treatments	Length (cm)	Surface Area (cm^2^)	Diameter (mm)	Volume (cm^3^)
Control	3418.27 ^c^	673.08 ^c^	0.64 ^b^	13.50 ^c^
Fertilizer	3952.24 ^b^	910.09 ^a^	0.78 ^a^	18.99 ^ab^
*A. flavus* KKMP34	3943.44 ^b^	865.81 ^b^	0.75 ^a^	17.70 ^ab^
*T. pinophilus* PBMP28	3979.19 ^b^	868.38 ^b^	0.72 ^a^	18.97 ^bc^
*Tr. zelobreve* PBMP16	4252.58 ^a^	862.94 ^b^	0.82 ^a^	19.05 ^a^
*Trichoderma* sp. PBMP43	4260.40 ^a^	927.50 ^a^	0.83 ^a^	19.41 ^a^
F-test	**	**	**	**
%CV	7.86	10.25	12.75	10.27

Different letters indicate significant differences among values within the same column using the LSD test. **, Significant difference at *p* ≤ 0.01.

**Table 6:** Effect of endophytic fungi on the total phenolic compound (TPC), anthocyanin (TAC), antioxidant activities, and nutrient uptake content of Maled Phai rice at the harvest stage.

Treatments	TPC (mg·gallic eq·100 g^−1^ DW)	TAC (mg·Cy-3-G eq·100 g^−1^ DW)	Antioxidant (% Radical Scavenging)	Nutrient Uptake (mg·g^−1^)		
				N	P	K
Control	92.84 ^e^	33.06 ^c^	87.83 ^d^	0.68 ^d^	0.25 ^d^	1.37 ^d^
Fertilizer	111.61 ^cd^	45.09 ^b^	90.94 ^c^	1.84 ^ab^	0.89 ^a^	3.25 ^a^
*A. flavus* KKMP34	116.00 ^bc^	47.09 ^b^	97.49 ^a^	1.59 ^c^	0.54 ^c^	2.65 ^c^
*T. pinophilus* PBMP28	110.39 ^d^	45.09 ^b^	91.80 ^b^	1.61 ^c^	0.52 ^c^	2.56 ^c^
*Tr. zelobreve* PBMP16	122.32 ^a^	55.77 ^a^	98.07 ^a^	1.93 ^a^	0.67 ^b^	3.18 ^a^
*Trichoderma* sp. PBMP43	119.86 ^a^	53.10 ^a^	93.25 ^a^	1.76 ^b^	0.66 ^b^	2.94 ^b^
F-test	**	**	**	**	**	**
%CV	9.01	16.53	4.06	27.70	28.64	24.58

Different letters indicate significant differences among values within the same column using the LSD test. **, Significant difference at *p* ≤ 0.01.

## Data Availability

Not applicable.

## Supplementary Materials

The following supporting information can be downloaded at: https://www.mdpi.com/article/10.3390/jof9090937/s1, Table S1: Correlation between endophytic fungi and % colonization with plant parameters of rice at harvest stage.
