# Supplementary material for: The Potential of Endophytic Fungi for Enhancing the Growth and Accumulation of Phenolic Compounds and Anthocyanin in Maled Phai Rice (Oryza sativa L.)
Source: J Fungi (Basel). 2023 Sep 16;9(9):937. doi: 10.3390/jof9090937 (PMC10532753; doi:10.3390/jof9090937)
Supplement: Supplementary file 1 [file jof-09-00937-s001.zip › jof-2610777-supplementary.pdf]

Table S1. Correlation between endophytic fungi and % colonization with plant parameters of rice at harvest stage.

Correlation	Root colonization	Height	No. of tillers	Total chlorophyll	Pn	Sc	Tr	WUE	No. of panicles	Total seed weight	Total shoot weight	Root FW	Root DW	TAC	TPC	Antioxidant	N	P
Height	0.93**																	
No. of tillers	0.42ns	0.81ns																
Total chlorophyll	0.67**	0.83**	0.23ns															
Pn	0.88**	0.85**	0.06ns	0.80**														
Sc	0.88**	0.80**	-0.124ns	0.60**	0.91**													
Tr	0.83**	0.82**	-0.047ns	0.72**	0.84**	0.77**												
WUE	0.24ns	0.22ns	0.27ns	0.29ns	0.47ns	0.40ns	-0.08ns											
No. of panicles	-0.14ns	0.65ns	0.90**	0.38ns	0.13ns	-0.14ns	0.58ns	0.23ns										
Total seed weight	0.62**	0.79**	0.45ns	0.78**	0.73**	0.67**	0.65**	0.92ns	0.50**									
Total shoot weight	0.08ns	0.25ns	0.68**	0.53*	0.37ns	0.16ns	0.07ns	0.55*	0.65**	0.63**								
Root FW	0.19ns	0.34ns	0.70**	0.42ns	0.38ns	0.29ns	0.32ns	0.26ns	0.60**	0.65**	0.46ns							
Root DW	0.01ns	0.21ns	0.89**	0.41ns	0.23ns	0.05ns	0.11ns	0.30ns	0.88**	0.61**	0.73**	0.69**						
TAC	0.68**	0.84**	0.29ns	0.91**	0.75**	0.54*	0.66**	0.28ns	0.43ns	0.86**	0.60**	0.45ns	0.54*					
TPC	0.72**	0.83**	0.41ns	0.89**	0.81**	0.62**	0.77**	0.25ns	0.49*	0.88**	0.50*	0.62**	0.56*	0.89**				
Antioxidant	0.78**	0.75**	0.12ns	0.60**	0.67**	0.53*	0.77**	0.18ns	0.21ns	0.58*	0.10ns	0.35ns	0.31ns	0.67**	0.79**			
N	0.51*	0.67**	0.66**	0.81**	0.67**	0.49*	0.56*	0.37ns	0.70**	0.90**	0.73**	0.66**	0.77**	0.86**	0.88**	0.54*		
P	-0.06ns	0.17ns	0.93**	0.49*	0.22ns	-0.03ns	0.11ns	0.29ns	0.92**	0.63**	0.79**	0.71**	0.94**	0.56*	0.58*	0.21ns	0.85**	
K	0.39ns	0.57*	0.75*	0.76**	0.58*	0.38ns	0.49*	0.31ns	0.77**	0.88**	0.76**	0.70**	0.83**	0.82**	0.85**	0.49*	0.98**	0.88**

**, Significant difference at P≤0.01; *, Significant difference at P≤0.05; ns, not significant
